# Global scientific trends on matrix metalloproteinase and osteosarcoma: A bibliometric and visualized analysis

**DOI:** 10.3389/fonc.2023.1064815

**Published:** 2023-02-06

**Authors:** Xin Wu, Shiwei Ma, Zhongguang Wu, Qiangqiang Zhao

**Affiliations:** ^1^ Department of Spine Surgery, Third Xiangya Hospital, Central South University, Changsha, China; ^2^ Department of Anesthesiology, Third Xiangya Hospital, Central South University, Changsha, China; ^3^ Department of Laboratory Medicine, Shenzhen University General Hospital, Shenzhen, China; ^4^ Department of Hematology, The Qinghai Provincial People’s Hospital, Xining, China

**Keywords:** matrix metalloproteinase, osteosarcoma, bibliometric, pulmonary metastasis, prognoses

## Abstract

**Objective:**

This study aimed to identify author, country, institutional, and journal collaborations and their impacts, assess the knowledge base, identify existing trends, and uncover emerging topics related to the role of Metalloproteinase in osteosarcoma.

**Methods:**

945 Articles and reviews associated with the role of Metalloproteinase in osteosarcoma were obtained from the WoSCC and analyzed by Citespace and Vosviewer.

**Results:**

The main aspects of research on the role of MMP in OS are invasion and metastasis. The latest hotspots were found to be the mechanism of MMP promoting invasion and metastasis, lung metastasis, and antitumor activity. Notably, invasion, metastasis, and antitumor activity were potentially turning points in the MMP-OS field. In the future, the primary research hotspot in the field of MMP-OS may be to study the mechanism, explore their role in the OS lung metastasis, and determine their role in the cancer therapy process.

**Conclusion:**

This study thus offers a comprehensive overview of the MMP-OS-related field using bibliometrics and visual methods, which will provide a valuable reference for researchers interested in the field of MMP-OS.

## 1 Introduction

Osteosarcoma(OS) is a relatively rare mesenchymal tumor but is the most common primary malignant bone tumor in young people and the fifth most common cancer in people under 25 years of age (1). OS usually occurs in long, weight-bearing bones, with the distal femur (43%), proximal tibia (23%), and humerus (10%) being the most common sites of significant pain and swelling of the affected bone as essential features (2), and OS can cause pathological fractures in some cases (3). The clinical character of OS is an aggressive invasion and highly metastatic cancer that often spreads to the lungs (4), resulting in a 5-year overall survival rate of 70% (5). Diagnosis of OS is mainly based on symptoms of pain, tenderness, and swelling of the affected bone, which can be diagnosed by imaging and histopathological biopsy. In recent years, although the level of diagnosis and treatment of OS has been continuously improved; However, some patients with OS have a terrible prognosis due to their high rate of lung metastases (6, 7). Therefore, new markers of OS metastasis are essential to reduce mortality in patients with OS.

**Figure 1:**
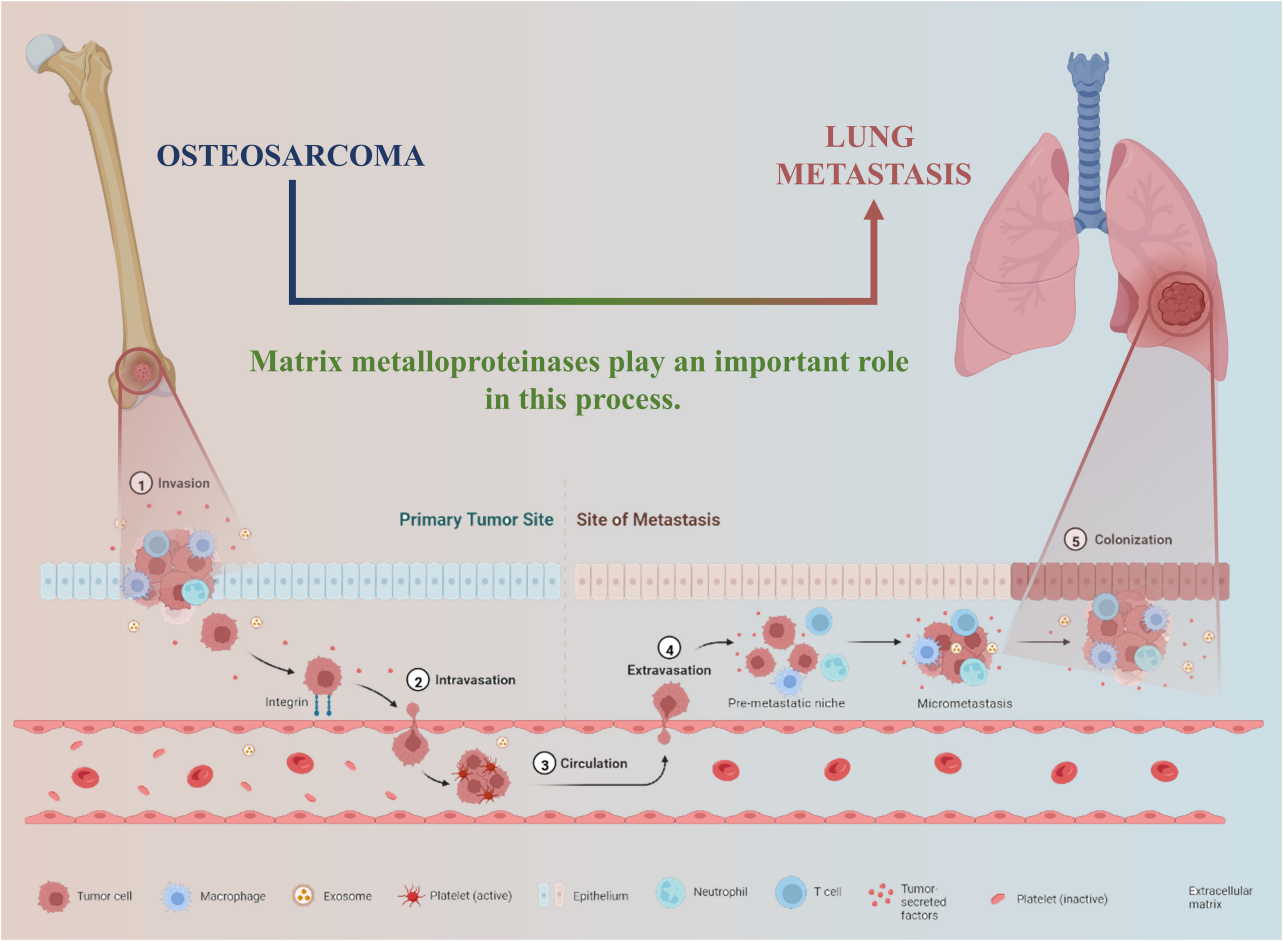
The emerging hot spot of the role of Metalloproteinase in OS may be to promote invasion and metastasis.

Matrix metalloproteinases (MMP) were first discovered by Gross and Lepiere when studying tadpoles in 1962 (8), and In 1992 Woessner introduced the biological properties of MMPs and matrix metalloproteinase tissue inhibitors (TIMPs) in their entirety (9). MMPs are a group of zinc and calcium-dependent proteolytic enzymes whose primary role is to hydrolyze the extracellular matrix, and TIMPs can inhibit their activity. At present, more than 30 kinds of MMPs have been discovered. In the 23 kinds of human MMPs, the basic structure includes signal peptides, propeptides, and catalytic domains, and there are other particular domains in some MMPs, such as (1) fibronectin repeat sequences (MMP-2, MMP-9); (2) Heme structure (except MMP-23); (3) Transmembrane domains (MMP-14, MMP-15, MMP-16, MMP-23, MMP-24), and so on. MMPs regulate a series of physiological processes and signal events, manipulate some bioactive molecules on the surface of cells, and change the biological behavior of cells, playing an essential role in the tumor microenvironment. Studies have shown that MMP affects the distant metastasis of cancer cell by affecting the invasion, migration, blood circulation, extravascular migration. (**Figure 1**) However, in most tumor microenvironments, the location and timing of MMPs exerting their proteolytic activity are unclear (10, 11).

Today, there are many ways to systematically review a field of study, among which the bibliometric analysis is one of the most commonly used methods. Bibliometric analysis is a proxy measure of the quality of research based on the frequency of citations as an indicator, which is a valuable tool for statistically evaluating the trend characterization of research results. Other methods, such as traditional reviews, meta-analyses, or experimental studies, cannot do this. Depending on strength, it is becoming increasingly important to assess research trends and develop guidelines. Provide a way to understand specific trends and rank academic groups and individuals by author, country, journal, citation, and publication date of the selected article (12). In addition, keywords that appear more frequently, including articles that have appeared in recent years and hot words, are analyzed to provide evidence to support future trends (13).

There is currently no similar analysis in the OS. This paper uses bibliometric analysis to describe the characteristics of the literature’s understanding of MMP in OS for nearly two decades and predict future research trends and hotspots. This study aims to objectively describe the areas of knowledge and new trends in MMP in OS research from the following four aspects, using two commonly used bibliometric tools, CiteSpace and VOSviewer (1). We intend to quantify and identify the general information of MMP in OS research by researching annual articles, journals, co-citation journals, countries, organizations, researchers, and co-cited researchers. (2) We strive to identify and study the most cited articles through a co-cited literature analysis to evaluate MMP’s knowledge base in the OS. (3) Identify knowledge structure and hot spot evolution through keyword analysis and co-citation reference outbreak analysis. (4) At the same time, through the analysis of the first 100 articles and the journals, countries, and keywords of the total cited journals, combined with the analysis content of (3), the research content and possible new directions in the field of MMP-OS were further determined (4). Association between MMP and prognosis of OS.

## 2 Materials and methods

### 2.1 Data collection

We used the WoSCC database because it provides the full range of data bibliometric software needed and is considered the most potent database (14, 15). Data were acquired from the WoSCC database on November 29, 2022. We searched for TS = (“Osteosarcomas” OR “Osteosarcoma Tumor” OR “Osteosarcoma Tumors “ OR “Tumor, Osteosarcoma” OR “Tumor, Osteosarcoma” OR “Sarcoma, Osteogenic” OR “Osteogenic Sarcomas” OR “Sarcomas, Osteogenic” OR “Osteogenic Sarcoma”) AND TS = (“endopeptidase*” OR “matrilysin*” OR “collagenase*” OR “stromelysin*” OR “matrixin*” OR “gelatinase*” OR “RASI-1” OR “enamelysin*” OR “epilysin*” OR “metallopeptidase” OR “metalloproteinase*” OR “metalloprotein*” OR “transin*” OR “proteoglycanase*” OR “leukolysin*” OR “endometase*” OR “metalloelastase*” OR “PUMP-1” OR “macrophage elastase*” OR “MMP*” OR “metalloprotease*” OR “ADAM protein” OR “serralysin*” OR “astacin*” OR “ADAM-” OR “ADAM1*” OR “ADAM2*” OR “ADAM3*” OR “ADAM4*” OR “ADAM5*” OR “ADAM6*” OR “ADAM7*” OR “ADAM8*” OR “ADAM9*” OR “ADAM0*” OR “ADAMT*” OR “RECK*”) We obtained two parts of the data: (1) from WoSCC all articles published from the establishment of WoSCC until November 29, 2022. (2) Get the top 100 most cited articles. Search Criteria: Language is limited to English; article type is limited to articles and reviews. The results are downloaded from the record content of Full-Text Records and Citations as Plain Text. Subsequently, we renamed the file for subsequent analysis, as CiteSpace can only identify the file in the form of “download*.” txt.” The language was limited to English.

### 2.2 Data analysis and visualization

Currently, the most commonly used programs in bibliometrics are VOSViewer, CiteSpace, SCI2, NetDraw, and HistCite. Given their characteristics and advantages, there is no consensus on the best software approach in bibliometrics. This study used VOSviewer and CiteSpace (16, 17). We use VOSviewer 1.6.15 to identify essential journals, commonly cited journals, researchers, co-cited researchers, and related knowledge graphs based on bibliographic information (18). In addition, we created keyword co-occurrences and clustering charts based on text data. First, we cleaned up the data. For example, “Mirabello, Lisa” and “ Mirabello, l” were merged in the authors’ analysis, and “matrix metalloproteinase” and “metalloproteinases” were unified into “MMP” in the keyword analysis. Second, we used the fraction counting method to set the maximum number of study participants per document to 25. The difference between the complete count and the parts count is the strength of the link. The score count calculates link strength by splitting articles by weight. If four researchers jointly publish an article, the strength of each link is calculated as 1/4 of the fractional count. For fractional counts, it is counted as 1. The performance of the fraction counting method in the author’s analysis is more reasonable, and the data obtained by the fraction counting method are more reasonable and more straightforward. The project’s other thresholds (T) are set according to different situations and annotated in the relevant tables and illustrations.

CiteSpace was a bibliometric and visual analysis tool developed by Professor Chen Chaomei, who specializes in exploring collaborations, key points, internal structures, potential trends, and dynamics in a particular field (19). Therefore, we used CiteSpace 5.7 to study and visualize co-occurrences of countries and organizations, dual journal graphs, high-frequency keyword trends, co-cited references, and citation bursts.

Before the investigation, the data is cleaned; For example, in the national analysis, articles from Taiwan are classified as China, while articles from England, Scotland, Northern Ireland, and Wales are classified as the United Kingdom. Similarly, we combined synonyms such as “MMP” and “ matrix metalloproteinase, “MMP inhibitors,” and “ matrix metalloproteinase inhibitor, “Central South University,” and “Central South University.” CiteSpace was set up as follows: Time Span, 1999-2022; year per piece, 1, pruning, minimum spanning tree and pruning slice network; selection criteria, Top N=50; Other companies follow the default setting.

We used Microsoft Office Excel 2019 to process articles published in the database every year. Obtained journal citation report published by IF and JCR divisions from Web of Science on March 22, 2022.

### 2.3 Prognostic value of MMPs in OS

The gene expression (RNA-Seq) was download from TARGET-OS dataset (https://ocg.cancer.gov/programs/target). We created an mRNA matrix using R and processed the transcriptome data to perform gene ID conversion with the corresponding script. We then compared the high- and low-expression survival curves by R software.

## 3 Results

### 3.1 The annual growth trend and the annual number of cited departments

**Figure 2:**
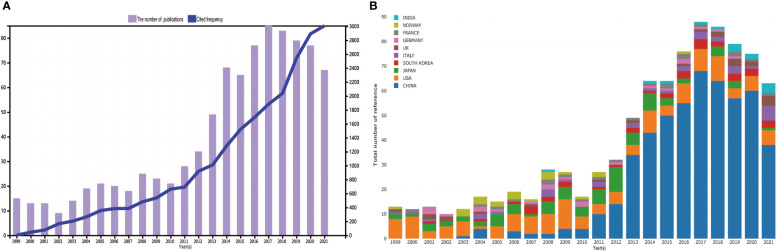
**(A)** Annual publication number and citation frequency of MMP-OS related studies. **(B)** Composition ratio of the top 10 countries by publication number.

Regarding data acquisition methods, we limited the article types to articles and reviews, and the language was English, and a total of 968 articles were obtained. The 968 eligible articles published between 1999 and 2022 were finally selected (**Supplementary Table 1**). **Figure 2A** shows that the literature related to matrix metalloproteinase and OS has steadily increased yearly. In addition, we analyzed the top 10 countries in terms of the number of articles published (**Figure 2B**): The United States was the most published country until 2010 (1999-2010). However, since 2011, China has shown great interest in matrix metalloproteinases and osteosarcoma, surpassing the United States to become the country with the most significant number of articles.

### 3.2 Journals and co-cited journals

**Figure 3:**
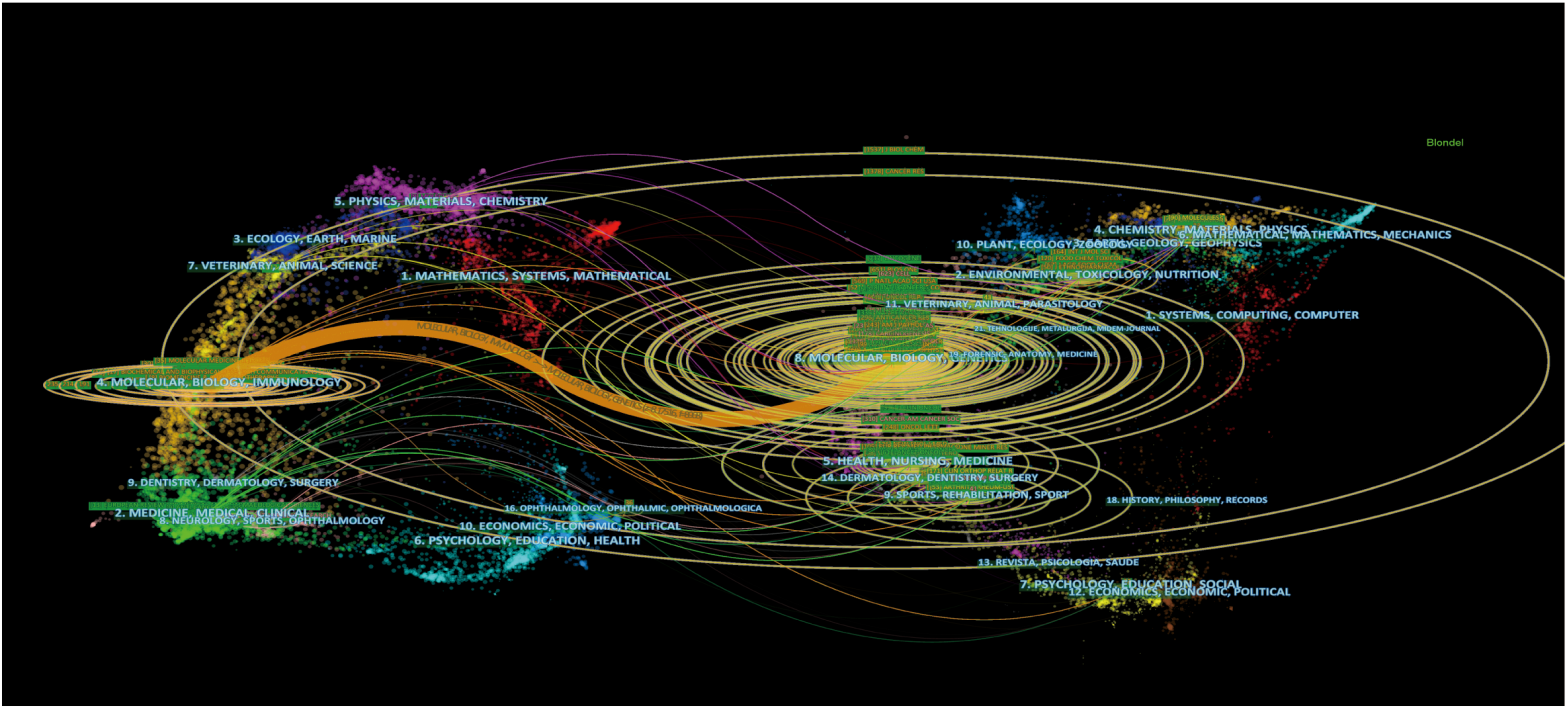
The dual-map overlay of journals related to MMP-OS research Notes: The citing journals were at left, the cited journals were on the right, and the colored path represents citation relationship.

We used VOSviewer to analyze journals and co-cited journals of the most significant and essential journals in the field of MMP OS. The results showed that 968 articles related to the field were published in 359 journals. MOLECULAR MEDICINE REPORTS published the most articles (35, 3.638%), followed by ONCOLOGY REPORTS, ONCOLOGY LETTERS, INTERNATIONAL JOURNAL OF ONCOLOGY, and TUMOR BIOLOGY (**Supplementary Table 1**). The United States and GREECE each account for 4/10. Three of the top 10 journals are from the Q2 JCR sector, and two impact factors (IF) are above 5 (**Supplementary Table 1**). Of the 3 363 commonly cited journals, 15 were cited > 400 times. As shown in **Supplementary Table 2**, the JOURNAL OF BIOLOGICAL CHEMISTRY has the highest frequency of citations (1537), followed by CANCER RESEARCH, ONCOGENE, PLoS One, and CELL. In front, ten co-cited journals and six from the JCR Q1 department. Notably, the United States occupies a part of the 80%. The double map overlay of the journal reflects the distribution of topics in the journal (**Figure 3**) (20). Citation journals are on the left and the cited journals on the right, and the color path indicates the citation relationship. Only an orange primary citation path is identified, which means published in Molecular/biology/immunology journals are primarily cited in research Published in the Journal of Molecular/Biology/Genetics.

### 3.3 Countries/regions and institutions

**Figure 4:**
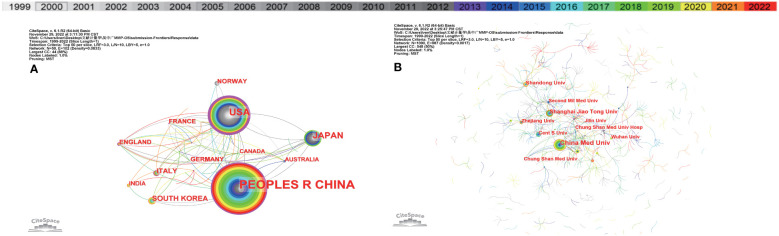
The co-occurrence map of **(A)** countries/regions and **(B)** institutions in MMP-OS research (T≥19). The size of node reflects the co-occurrence frequencies, and the links indicate the co-occurrence relationships. The color of node and line represents different years, colors vary from purple to red as time goes from1999 to 2022; and node with purple round means high betweenness centrality (>0.1).

1098 research institutes from 50 countries published 945 articles. The largest number of publications is from China (542, 48.61%), followed by the United States (146, 13.09%), Japan (77, 6.91%), and South Korea (36,3.23%) (**Supplementary Table 3**). Certain nodes points, such as the United States, China, Italy, the United Kingdom, and France, present a purple circle of high intermediary centers (≥0.10) (**Figure 4A**), which is generally considered to be an important turning point that could lead to revolutionary discoveries, as a bridge. In addition, according to the link color, the United States (1999), Japan (1999), Italy (1999), Norway (1999), the United Kingdom (1999), Canada (1999) were the first countries to study MMP and OS. We use minimal spanning tree pruning to clarify the network (**Figure 4A**). The map of unpruned countries contains 50 nodes and 102 links at a density of 0.0833, showing active cooperation between countries.

The top 10 institutions are all from China (10/10) (**Figure 4B**) (**Supplementary Table 3**). China Medical University (52, 2.53%) published the most papers, followed by Shanghai Jiao Tong University (28, 1.36%), Shandong University (22, 1.07%), Central South University (19, 0.93%), Chung Shan Medical University (18, 0.88%), Zhejiang University ((18, 0.88%), Chung Shan Medical University Hospital (17, 0.83%) and Jilin University/Wuhan University/Second Military Medical University (16, 0.78%) (**Supplementary Table 3**).

### 3.4 Author and co-cited author

**Figure 5:**
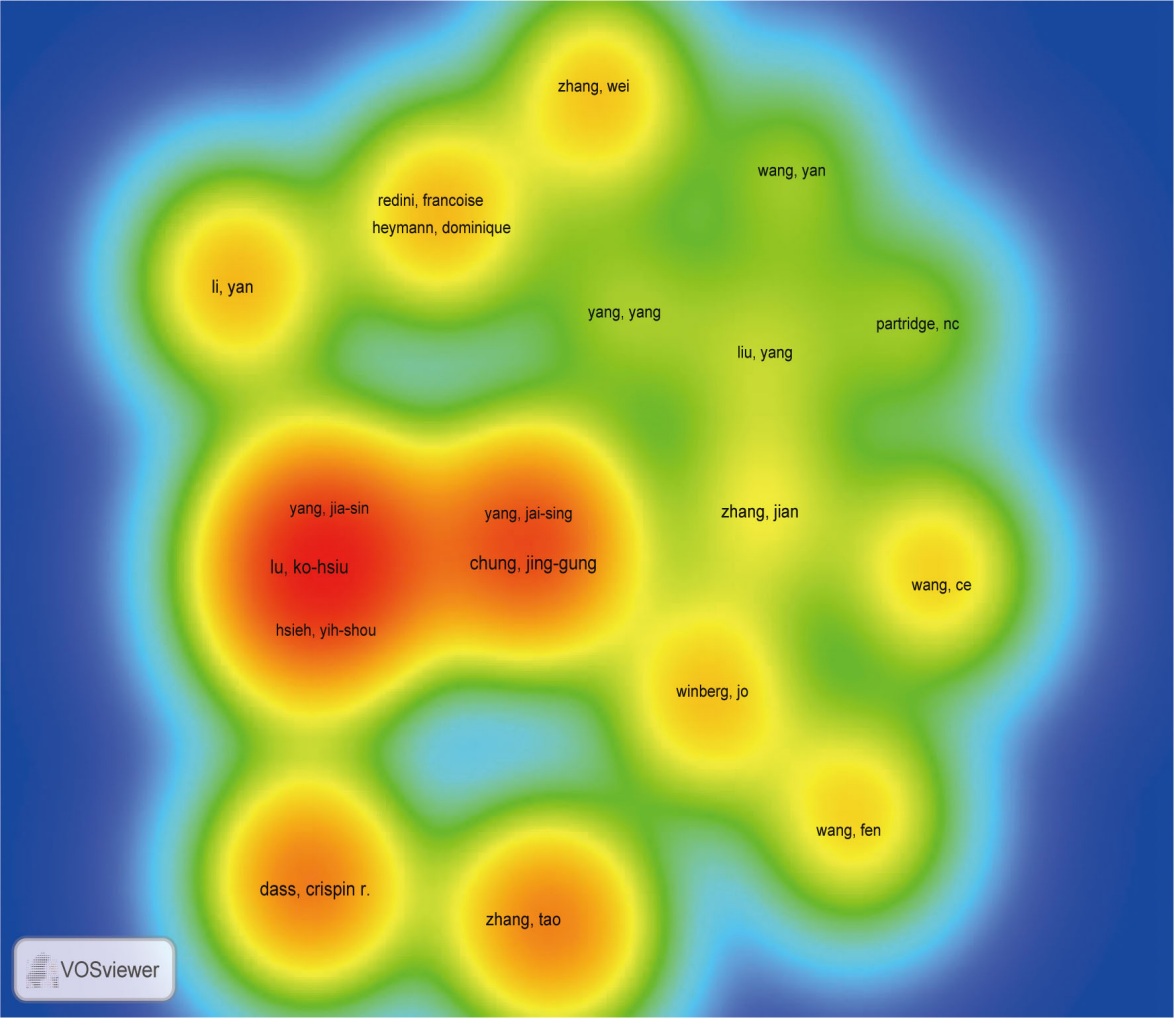
The density map of authors in MMP-OS research (T≥5). The size of word, the size of round, and the opacity of yellow is positively related to the publication frequency.

A total of 5294 researchers were involved in MMP and OS. 32 researchers published more than five papers. Lu, Ko-Hsiu. wrote the most articles (n = 29), followed by Yang, Shun-Fa (n = 27), Yang, Jia-Sing (n = 17), Hsieh,Yih-Shou (n = 16), Hsieh, YI-HSIEN (n = 13), Chen, Pei-Ni (n = 11), Chung, Jing-Gung (n = 9), Yang, Jai-Sing (n = 7), Chueh, Fu-Shin (n = 7), Dass, Crispin R and Choong, Peter F. M (n = 6) (**Supplementary Table 4**). We screened authors (n =60) who had published more than four papers (T≥4) to construct an author network diagram (**Figure 5**).

The knowledge graph presents high-frequency researchers. As can be seen from **Figure 5**, lu ko-hsiu, yang Jia-sin, yang Jai-sing, yang shun-fa, tang Chih-Hsin, and Chung jing-gung are closely linked, forming the darkest group of authors, indicating that this group has made outstanding contributions in the field of MMP-OS. Zhang tao constitute the second largest group of writers. dass Crispin r was the third largest group of writers.

**Figure 6:**
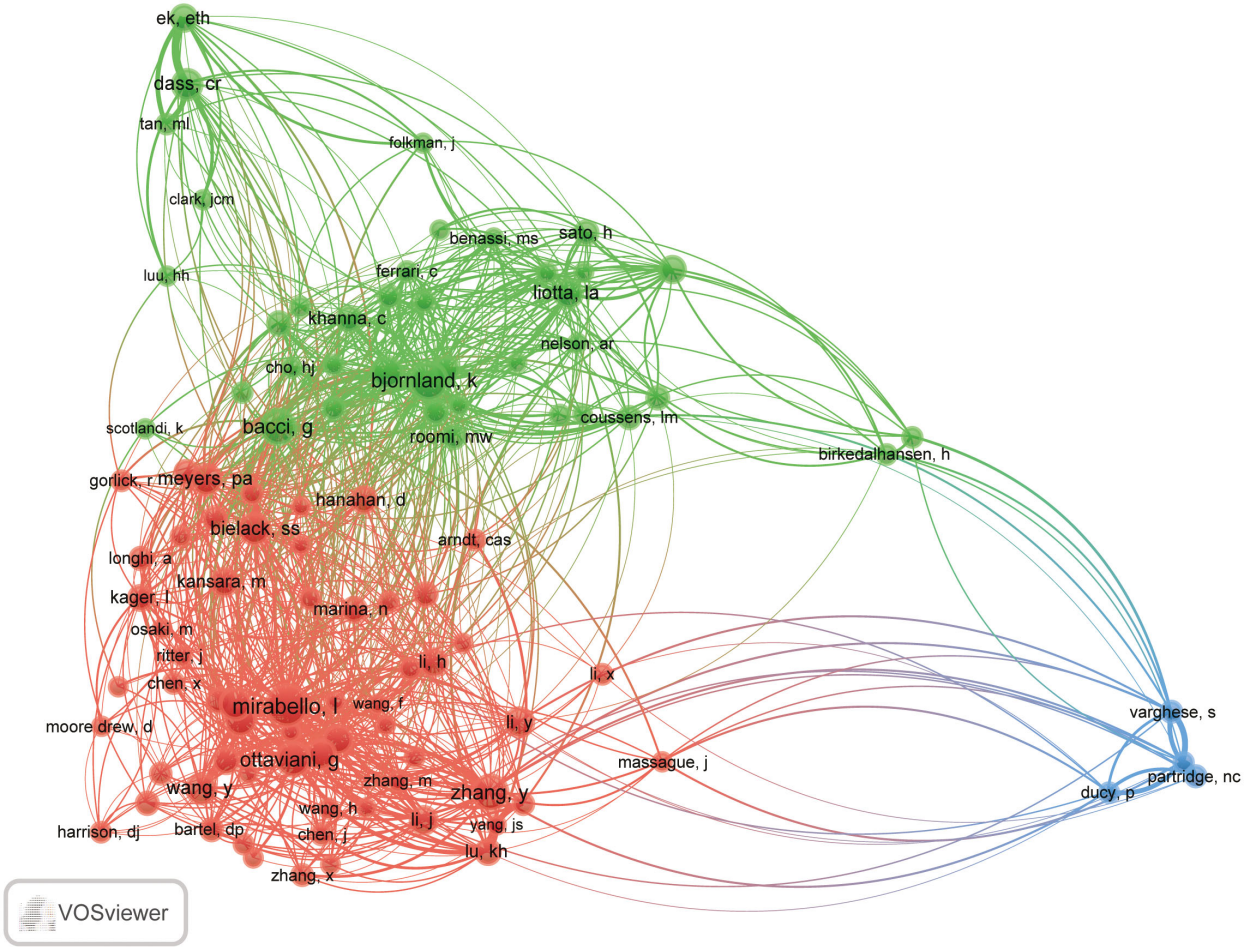
The co-occurrence map of co-cited authors in MMP-OS research (T≥40). the link indicate the co-occurrence relationship between authors, and the same color of node represent the same cluster.

Researchers have been co-cited in a series of publications (21). Of the 22641 researchers who were co-cited, one was cited more than 100 times. No. 1 was Mirabello, Lisa (108), followed by Bjornland, Kristin (90), Bacci, G. (80), Ottaviani, G. (74), and Zhang, Yuanyuan (76). The co-citation frequency of the first five authors is between 58 and 64 (**Supplementary Table 4**), and the co-cited authors (n=95) and at least 20 co-cited authors (T≥20) are selected to draw a network map of co-cited researchers (**Figure 6**), and the same color indicates the same cluster. The co-cited authors are divided into three main groups. In the field of MMP-OS, authors of the same cluster, such as Mirabello l, Ottaviani g, zhang y, and bielack, ss work closely together. At the same time, we also observed close collaboration between different clusters such as Mirabello l, bjornland k, dass cr. varghese s, and partridge nc. Notably, Mirabello, Lisa appears not only in high-frequency authors but also in highly cited scholars, suggesting that he made an essential contribution to the field.

### 3.5 Keyword co-occurrence, clustering, and development

**Figure 7:**
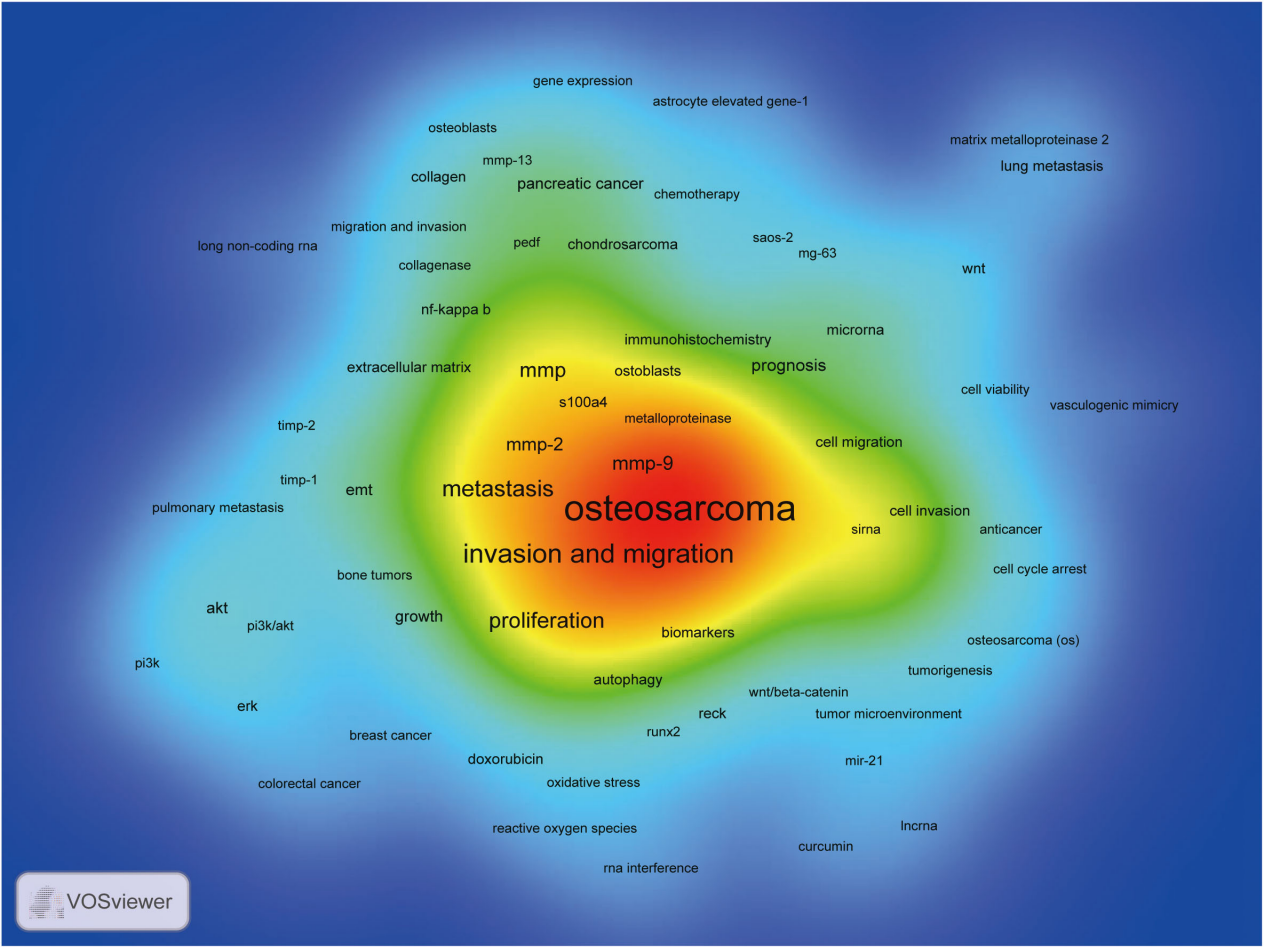
The density map of terms in MMP-OS research (T≥6), The size of word, the size of round, and the opacity of red is positively related to the co-occurrence frequency.

**Figure 8:**
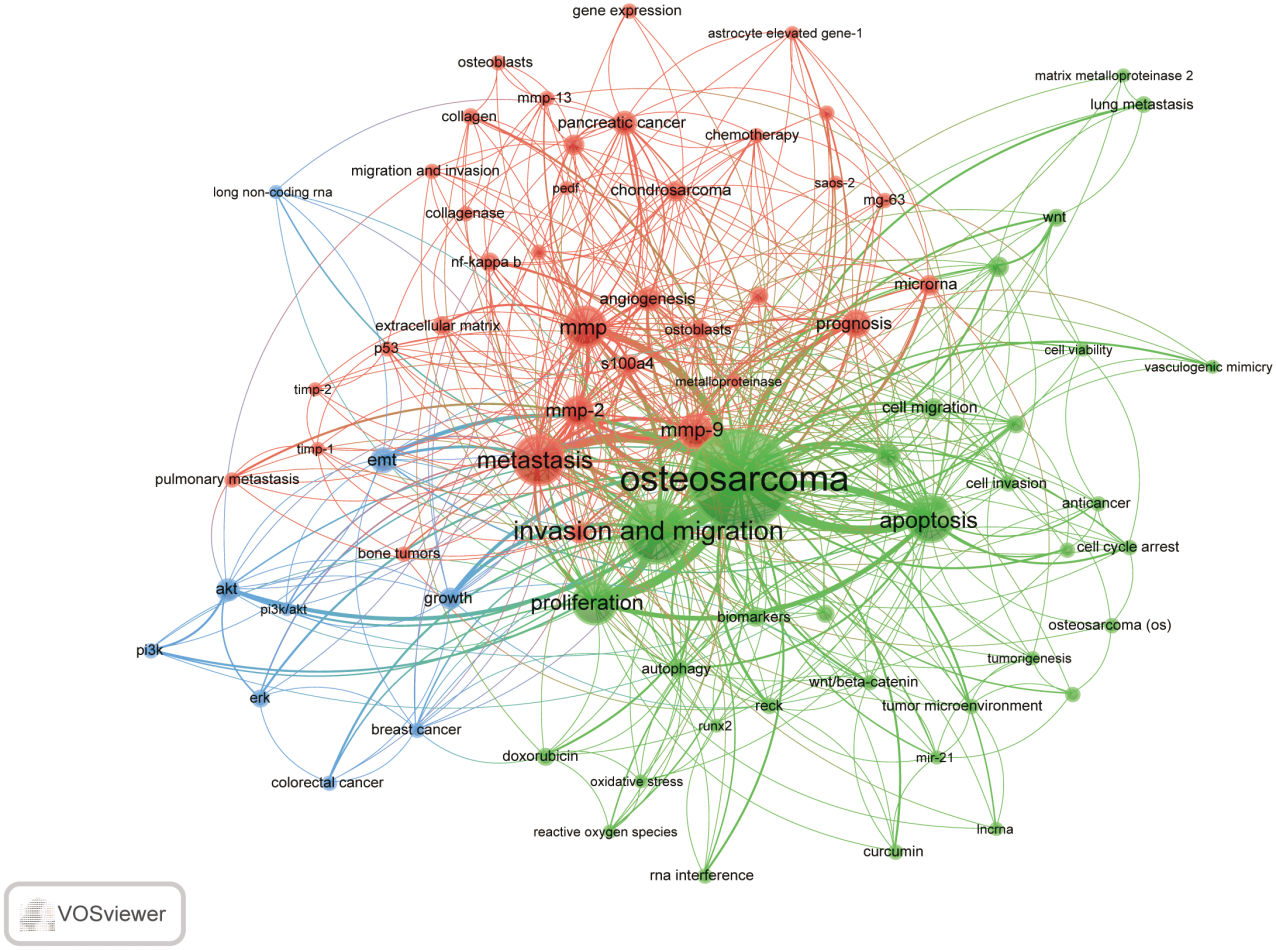
keyword co-occurrence network and clusters in MMP-OS research (T≥6, contains 72 items, 3 clusters and 3708 links; maxlines = 1000.). The size of node and word reflects the co-occurrence frequencies, the link indicate the co-occurrence relationship, and the same color of node represent the same cluster.

**Figure 9:**
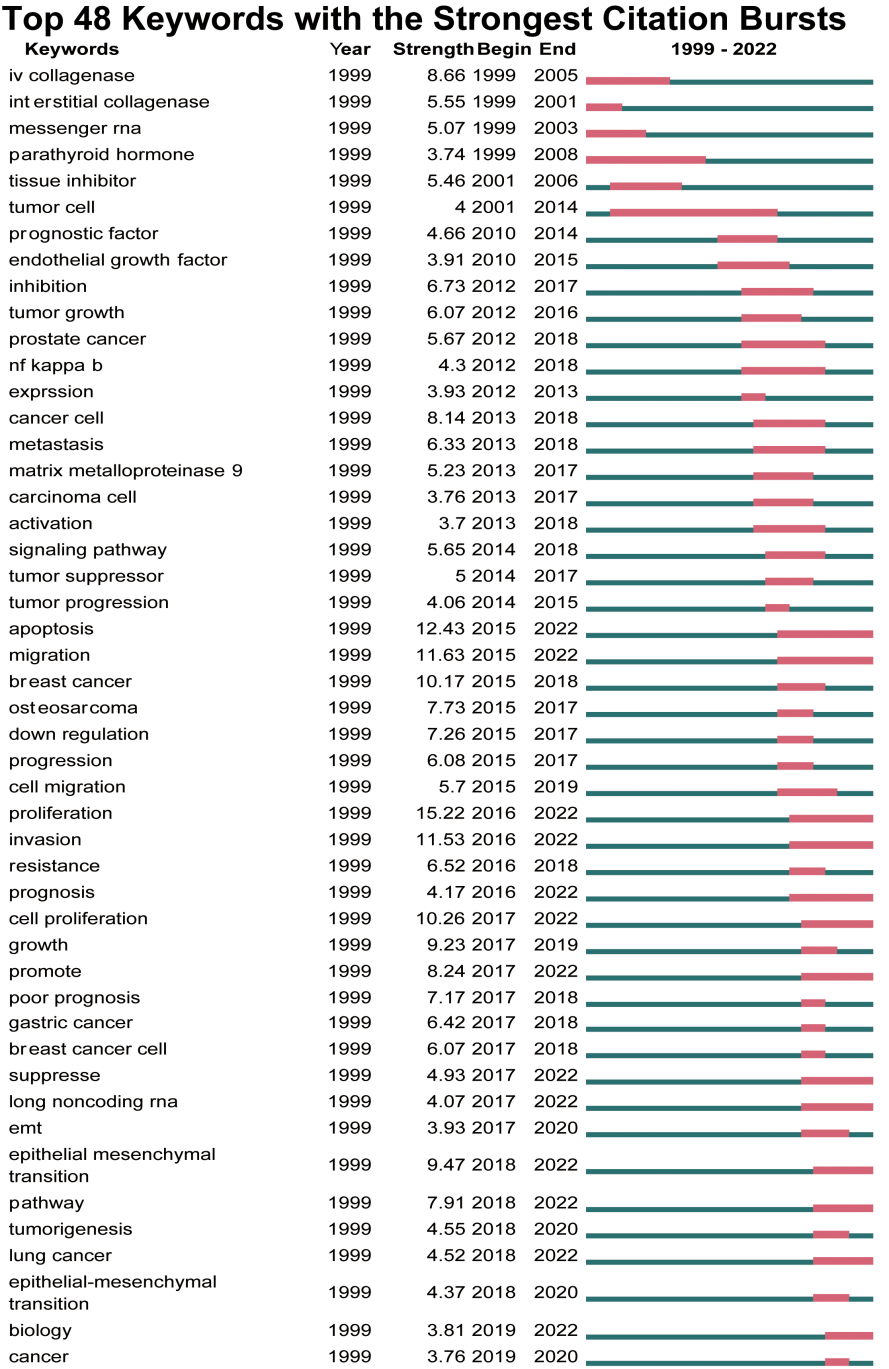
Keywords with strongest citation burst’s view of MMP-OS research.

VOSviewer was used for keyword co-occurrence (**Supplementary Table 5**; **Figures 7**-**9**) and cluster analysis (**Figure 7**). A total of 3893 keywords were extracted, of which 302 keywords appeared more than 5 times, and 246 keywords appeared more than 6 times. Keyword density maps (**Figure 7**) can find high-frequency co-occurrence entries, revealing hot spots in specific academic fields. **Supplementary Tables 5**, **6** show that OS is a fundamental term, occurring 491 times, followed by invasion and migration, metastasis, apoptosis, proliferation, mmp, mmp-9, mmp-2, prognosis, and EMT.

Cluster analysis can reveal the knowledge architecture of a research area (22). Divide the network into three clusters based on the link strength of the keyword (**Figure 8**). Keywords in each cluster are highly homogeneous. Cluster 1 (red) 34 co-occurrence keywords, including mmp, mmp-9, metastasis, progression. The keyword for cluster 1 is mainly related to the function of MMP in OS. Cluster 2 (blue) is mainly related to the role of MMP in OS and includes 25 keywords, such as EMT, breast-cancer, growth and PI3K/AKT. Cluster 3 (green) focuses on MMP-OS and contains 13 keywords, including OS, invasion and migration, proliferation, apoptosis.

Keyword bursts are words that frequently appear in literature published over time. At CiteSpace, we set the outbreak time to ≥ 2 years. We identified 20 groups of keywords with a frequency greater than 5, and we selected the top 48 keywords for object analysis. As can be seen from **Figure 9**, the top 48 burst keywords were published after 1999, and there were high citation events in 2017 (9/48, 18.75%), followed by 2015 (7/48, 14.68%), 2013, and 2012 (5/48, 10.4%). It is worth noting that by 2022, 13 articles (27.08%) will be in a state of an outbreak. The keyword with the strongest burstiness (strength=8.66) was entitled “IV collagenase” with co-occurrence bursts from 1999 to 2015.

### 3.6 Co-citation references and reference burst

We used CiteSpace to identify co-cited literature. (**Supplementary Table 7**) The results are displayed that the top 10 cited articles have been cited ≥31, of which three references have been quoted more than 60 times. The most cited reference was a paper by Mirabello, Lisa et al. published in CANCER in 2009 entitled “OS Incidence and Survival Rates From 1973 to 2004 Data from the Surveillance, Epidemiology, and End Results Program”, followed by an article titled “The Epidemiology of OS” (21).

**Figure 10:**
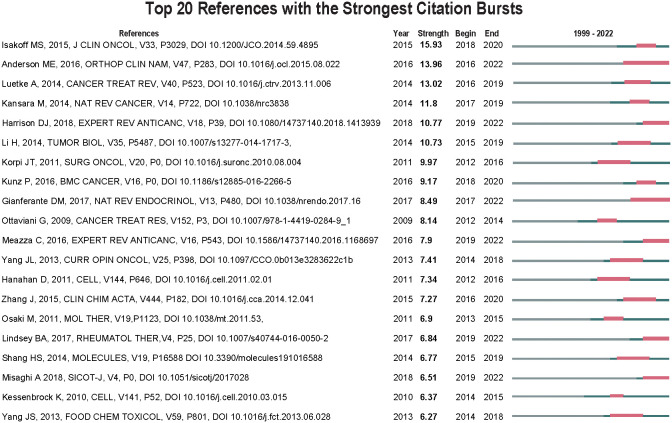
Top 20 references with the strongest citation bursts (sorted by the strength). The Blue bars mean the reference had been published; the red bars mean citation burstiness.

A reference burst is defined as a reference that is frequently referenced over a while. At CiteSpace, we set the burst duration to ≥ 2 years. We identified 113 of the most potent references and selected the top 20 works of literature as the subject of analysis. As can be seen from **Figure 10**, the top 20 literature published after 1999 had a high number of induced outbreaks in 2022 (6/20, 30%), followed by 2020 (3/20, 15%), 2019 (4/20, 20%), and 2018 (2/20, 10%). It is worth noting that by 2022, 6 literature (39.29%) are in a state of the outbreak. The most substantial reference to the outbreak is cited titled “ OS: Current Treatment and a Collaborative Pathway to Success” published in JOURNAL OF CLINICAL ONCOLOGY by Isakoff, Michael S, etc. in 2015, with citation bursts from 2018 to 2021 (2).

### 3.7 Analysis of journals, countries, and keywords of the top 100 most-cited articles

**Figure 11:**
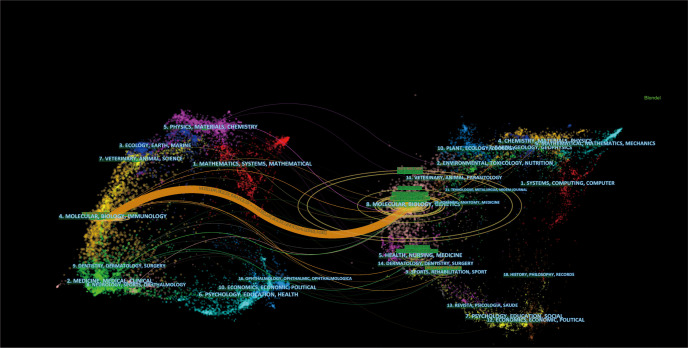
The dual-map overlay of journals related to top 100 most cited references in MMP-OS research. The citing journals were at left, the cited journals were on the right, and the colored path represents citation relationship.

The top 100 most-cited articles were defined as cited articles with a high correlation with MMP-OS (23). We analyzed the journals and co-cited journals of the top 100 most-cited articles. (**Supplementary Tables 8**, **9** and **Figure 11**) 19 journals have published more than two articles, of which JOURNAL OF BIOLOGICAL CHEMISTRY (n=7), ONCOTARGET (n=5), BMC CANCER (n=4), FOOD AND CHEMICAL TOXICOLOGY(n=4), ONCOLOGY REPORTS(n=4) and TUMOR BIOLOGY (n=4) ranks top three, followed by CANCER RESEARCH (n=3), CARCINOGENESIS (n=3) and CLINICAL & EXPERIMENTAL METASTASIS (n=3).

In the co-cited journals, the JOURNAL OF BIOLOGICAL CHEMISTRY (n=267) was the most common, followed by CANCER RESEARCH (n=245), ONCOGENE (n=125), NATURE (n= 84), PROCEEDINGS OF THE NATIONAL ACADEMY OF SCIENCES OF THE UNITED STATES OF AMERICA (n= 83) and CELL (n= 77). Consistent with the overall field analysis, journal analysis of the top 100 most-cited articles also identified only one primary orange citation path, which suggests that research published in the Molecular/Biology/Immunology journal is predominantly cited by research published in the Molecular/Biology/Genetics journal.

**Figure 12:**
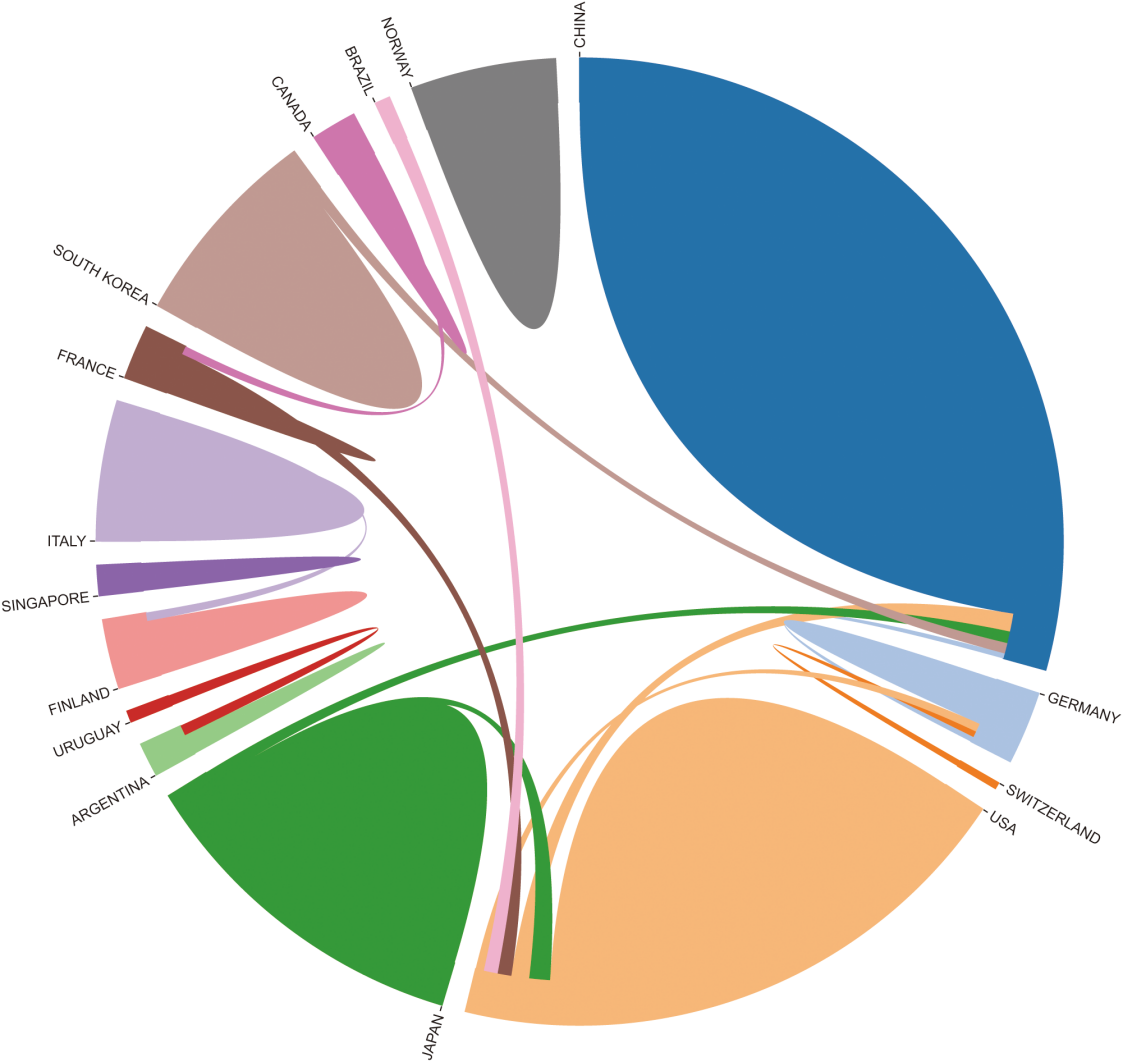
Country-to-country relations of Top 100 Most-cited Articles.

**Figure 13:**
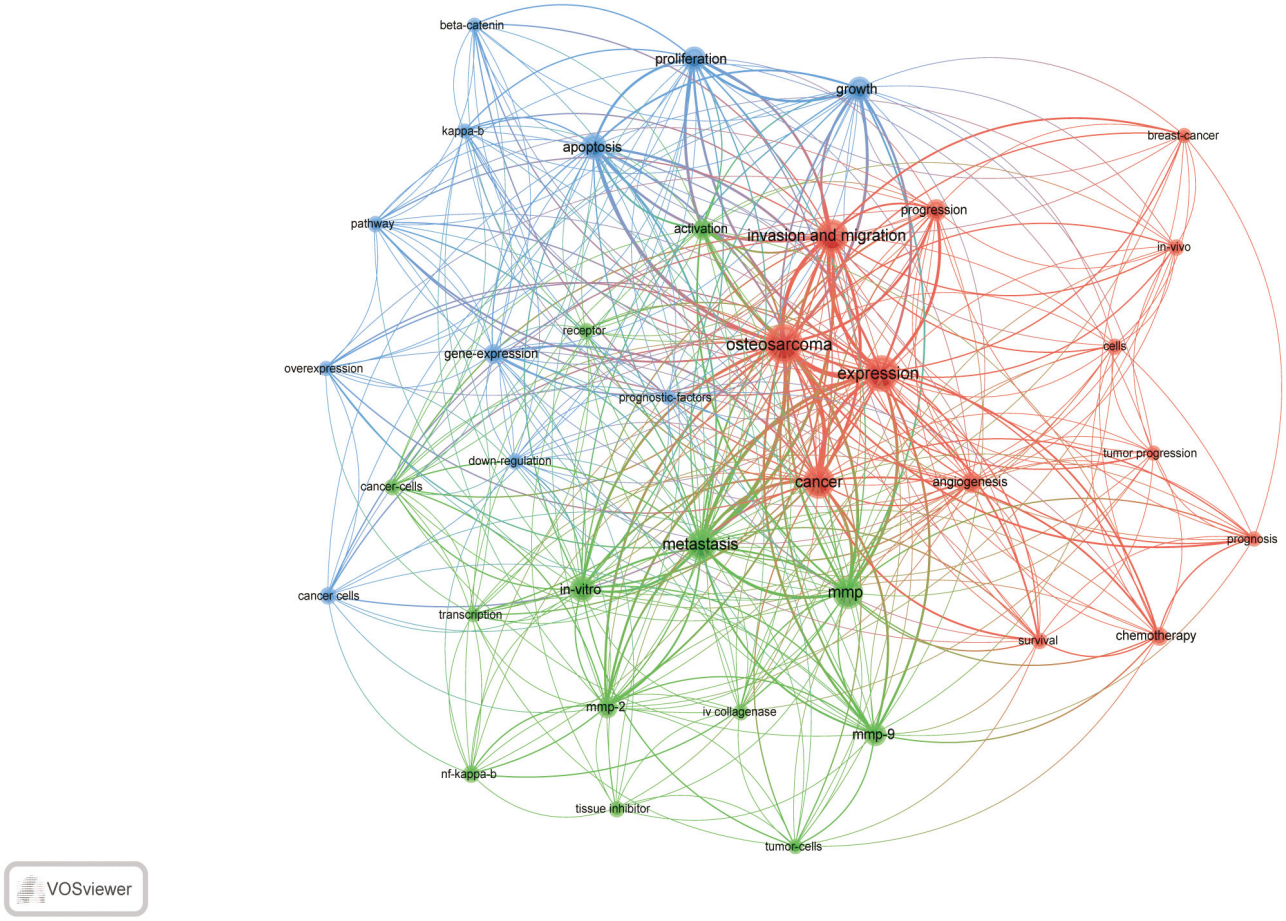
keyword co-occurrence network and clusters in MMP-OS research (T≥2, contains 37 items, 3 clusters and 788 links; maxlines = 1000.). The size of node and word reflects the co-occurrence frequencies, the link indicate the co-occurrence relationship, and the same color of node represent the same cluster.

We also analyzed the countries with the top 100 most-cited articles (**Figure 12**). China (n= 40) was the nation with the most papers, followed by the United States (n= 35). China has frequent academic exchanges with America, while the United States has ties with 16 countries, including JAPAN, ITALY, and SOUTH KOREA. In order to further analyze the development content and trend of the MMP-OS field, we analyzed the keywords of the top 100 articles. The keyword of the top 20 (**Supplementary Table 6**) coincided with the top 20 (**Supplementary Table 5**) in the MMP-OS field, such as invasion and migration, OS, apoptosis, metastasis, proliferation, mmp, cancer, growth, and activation. This shows that the primary research content of MMP-OS is related to the above keywords. The net was separated into three clusters according to the link strength of keyword co-occurrence (**Figure 13**). Cluster 1 (red) was the most significant cluster with 13 co-occurrence keywords, including OS, expression, invasion, and migration, cancer, progression, angiogenesis, chemotherapy, cells, *in-vivo*, and survival. The theme of Cluster 1 was the role of MMP in OS. Cluster 2 (green) was associated with the upstream molecules of MMP and contained 6 terms, including metastasis, mmp, *in-vitro*, mmp-9, mmp-2, activation, nf-kappa-b, cancer-cells, iv collagenase, receptor. Cluster 3 (blue) was primarily associated with the downstream molecules of MMP effects and included 13 terms, such as apoptosis, proliferation, growth, gene expression, cancer cells, down-regulation, pathway, beta-catenin, kappa-b, and overexpression.

### 3.8 Relationship between MMP and OS prognosis

**Figure 14:**
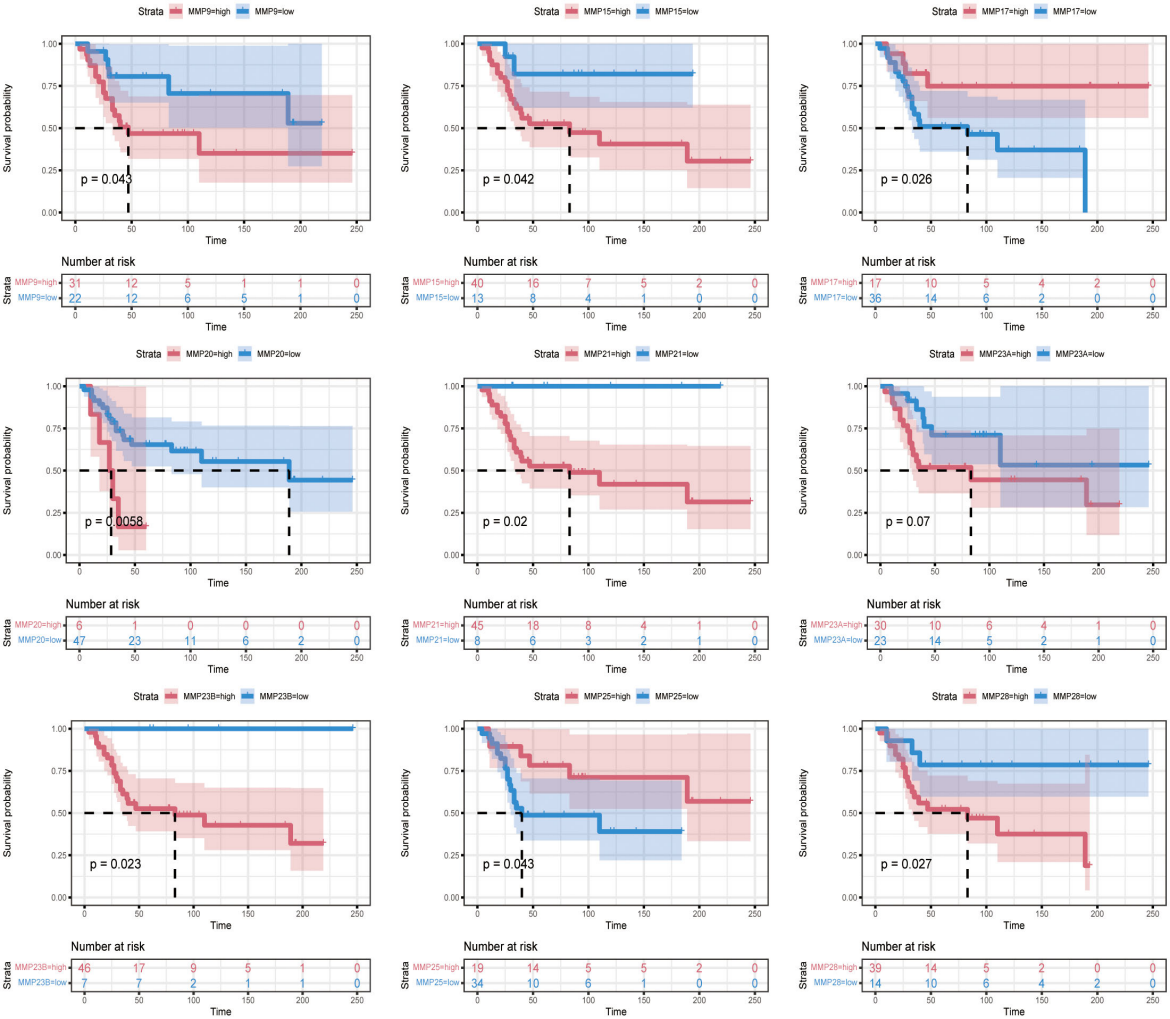
Association between the MMPs expression and the prognosis of OS.

To investigate the prognostic relationship between MMP and OS, we performed prognostic analysis. Kaplan–Meier survival curves indicated that the upregulated MMP9, MMP 15, MMP20, MMP21, MMP23A, MMP23B and MMP28 expression was remarkably associated with poor OS and the downregulated MMP17 and MMP25 expression was remarkably associated with poor OS (**Figure 14**). The relationship between the expression of other proteins of mmp and the prognosis of OS was illustrated in **Figures S1** and **Figure S2**.

## 4 Discussion

### 4.1 General information

According to data from the WoSCC database as of November 29, 2022, 4,599 authors from 1,097 institutions in 50 countries have published 968 studies on MMP-OS in 359 academic journals.

Annual production variation and frequency of citations are essential indicators for evaluating trends in this area (24). MMP-OS-related research officially began in 1999. Bjorn and K et al. proposed that MMP can facilitate the metastasis of OS cells (25). Since then, related articles in the MMP-OS field have shown an upward trend (**Figure 2**). MMP-OS-related articles can be divided into three stages: “budding,” “stable growth,” and “rapid development process.” “Sprout” (1999–2011): The role of MMP in OS began to be studied. For those 13 years, no more than 30 articles were written each year. “Steady growth” (2012-2017): The role of MMP in OS has attracted more and more attention from scientists, and production has increased steadily yearly. “Rapid Development Process” (2018 to present): The number of papers published every year has increased steadily; In addition, the frequency of citations of papers has also continued to increase, indicating that the role of MMP in OS has received more and more attention from researchers and has developed rapidly. In addition, the development trend in this field is up-and-coming.

The analysis of journals and co-cited journals (**Supplementary Tables 1**, **2**) revealed that the MOLECULAR MEDICINE REPORTS published the most significant number of studies on MMP-OS. At the same time, the JOURNAL OF BIOLOGICAL CHEMISTRY obtained the most significant number of co-cited references. Both are cell biology journals, consistent with graph analysis (**Figure 3**), and the journal’s dual-map overlay represents their thematic distribution status. This overlap shows a central citation path from the journals of molecular/biology/immunology to the journals of molecular/biology/genetics. At the same time, journals of JCR divisions in three zones and above took up most of the top 10 journals (77%) and co-cited journals (90%), indicating that these journals publish studies on and are critical for MMP and OS-associated research. The analysis of countries/institutions related to MMP-OS studies shows (**Supplementary Table 3**; **Figure 3**) that China, the United States, and JAPAN are the top three producers.

However, China, the United States, Japan, Italy, and South Korea could induce revolutionary breakthroughs. In addition, the United States, Japan, Italy, the United Kingdom, Norway, and Canada were the first to conduct MMP-OS-related research, followed by Germany and South Korea, among the top ten producers. This shows that the United States has been fruitful and influential in MMP-OS research. It is worth noting that China started relatively late, but in recent years it has become one of the most productive countries. This is consistent with the findings of bone tumors and may be associated with socioeconomic achievements and financial investments in academic research in these countries. In addition, cooperation between different countries, especially between the United States, is very positive, suggesting that the study of MMP-OS has attracted widespread attention worldwide, and the United States is the main center of cooperation. The top 10 institutions are all from China. In addition, our team also found fruitful collaborations between China Medical University, Shanghai Jiao Tong University, Shandong University, and other institutions, revealing their significant contributions to the field of MMP-OS.

Highlighting the contributions of active scholars, such as those who co-appear or co-cite articles in a particular field, can provide more direction and guidance to help researchers continue on this path. Here (**Supplementary Table 4**; **Figures 4**, **5**), Lu, Ko-Hsiu published the most papers, while Mirabello, Lisa was co-citing the most. At the same time, we found that Dass, Crispin R is not only the most fruitful author but also one of the top ten co-cited researchers, and Dass, Crispin R has made outstanding contributions in the field of MMP-OS. In addition, maps of researchers and co-cited researchers provide data from potential partners and solid academic teams. In the field of MMP-OS, scholars actively collaborate within and between organizations, especially among predominant researchers. A significant article titled “ Osteosarcoma Incidence and Survival Rates From 1973 to 2004 Data From the Surveillance, Epidemiology, and End Results Program” was co-cited 76 times (26). This suggests that the research of these powerful groups is widely followed and potential academic collaborators.

### 4.2 Knowledge base

Commonly cited references are those that have been cited together in different articles. However, the knowledge base is a collection of common citations by relevant research teams, not precisely highly cited literature. In this bibliometric analysis, the top 10 MMP-OS co-cited (**Supplementary Table 7**) are listed below.

In 2009, CANCER published the most co-cited MMP-OS studies by Mirabello, Lisa, et al. (n= 76) (26). This paper describes that the National Cancer Institute analyzed patients with OS between 1973 and 2004 and found that the incidence of OS was bimodal, more common in men, most often in the lower long bones, and survival rates have leveled off since the mid-1980s. Ottaviani, Giulia et al. wrote the second co-citation research on Cancer Treatment and Research (27). This study further illustrated the bimodal age distribution of OS: the first peak is in puberty, and the second peak is old. The first peak is in the 10-14 age group, coinciding with a growth spurt during puberty. This suggests a close relationship between adolescent growth spurts and OS. The second peak of OS occurs in adults over the age of 65. It is more likely to represent a second malignancy associated with Paget’s disease. The third co-cited article was published in the METHODS by Livak, KJ. (2001) (28). This article demonstrated that the most commonly used method in MMP in OS research is the real-time quantitative PCR experimental method.

The fourth co-cited paper was published in the JOURNAL OF SURGICAL RESEARCH by Bjornland, K. in 2005. This study reported that MMP molecules MMP significantly enhances OS cell invasion and migration by regulating proteolytic activity in OS. In 2002, Egeblad, M et al. published the fifth co-cited study in NATURE REVIEWS CANCER (29). This review summarizes previous studies and concludes that MMPs can promote cancer progression by increasing cancer cell growth, invasion and migration, metastasis, and angiogenesis, and concludes that MMP inhibitors could improve clinical efficacy by treating cancer. JOURNAL OF CLINICAL ONCOLOGY published the sixth article by Bielack, SS. in 2002 (30). This clinical trial of OS showed that tumor location and size, primary metastases, chemotherapy response, and surgical remission have independent prognostic value for OS. In 2012, CANCER TREATMENT REVIEWS published the seventh co-citation study by Luetke, Anja, et al (31).

Here, some experts were aware of the technical level of OS treatment at that time, and the prognosis of patients with advanced OS was getting better and better, but they also found that the problems and challenges brought by tumor aggressiveness to the treatment of OS may be a crucial influencing factor affecting the prognosis and progression of advanced OS. The eighth co-cited article was written by Marina, N et al. in ONCOLOGIST, 2004. This review summarizes the strategies for treating OS at the time and reviews its clinical features, imaging and laboratory tests, and pathological examination. The etiological and biological factors associated with the development of OS are discussed. It also looks forward to the future direction of the biological treatment of OS (32). In 1998, the Medical and pediatric oncology published the ninth co-citation research completed by Himelstein BP, Asada N, Carlton MR, and Collins MH (33). This study described that MMP-9 was highly expressed in OS samples before all treatments but decreased overall in all post-treatment samples. The significantly high expression of MMP-9 in metastatic lesions indicates that MMP-9 may promote OS metastasis. In 2015, the tenth co-cited paper was published in the JOURNAL OF CLINICAL ONCOLOGY by Isakoff, Michael S, and others (2). Here, they reviewed the tremendous progress made in curing more OS and improving patients’ quality of life over the past 40 years. They summarized the clinical trials at the time and suggested that some new therapies would emerge to improve the survival rate of OS.

Overall, the top 10 co-cited references focused on the review (published four times in 2002, 2004, 2014, and 2015), the epidemiological characteristics of OS (including morbidity characteristics, common incidence sites, age distribution, and etiology, etc.), the progress and problems of OS treatment (review of treatment strategies, the main challenge in treatment is metastatic tumors, etc.). The expression and function of MMP in OS (high expression, promoting metastasis).

### 4.3 Hot topic development, knowledge structure, and emerging topics

In bibliometric analysis, keyword/term co-occurrence rates (**Supplementary Table 5** and **Figure 7**) can represent hotspots in an academic field (34), and keywords with the most powerful citation bursts (**Figure 9**) can show the new hotspots. High-frequency keywords (**Supplementary Table 5** and **Figure 8**), including OS, invasion and migration, metastasis, apoptosis, proliferation, MMP, MMP-9, MMP-2, and prognosis, are considered the focus of MMP-OS research. Keywords presenting strongly-cited breakthroughs (**Figure 9**) could also characterize the new topics of a field (35–37). The intensity of proliferation burstiness in 1999 was the strongest (intensity = 15.22, 2016-2022), indicating that MMP may play a role in OS primarily by regulating proliferation. The key word with the second strongest outbreak intensity is apoptosis (intensity = 12.43, 2015-2022), suggesting that MMP may also play a role in OS mainly by modulating apoptosis. The following keywords for intensity are migration (intensity = 11.63, 2015-2022), and invasion (intensity = 11.53, 2016-2022), suggesting that MMP can promote the occurrence of EMT in OS, which leads to tumor and migration and migration. The intensity of IV collagenase burst in 1999 was 8.66 (1999-2005), indicating that IV collagenase is an earlier and more studied type of MMP in OS. Considering their high centrality, the invasion and migration may serve as a turning point and are also likely to develop into future research hotspots in MMP-OS (38, 39).

Unfortunately, while most studies focus on the role of MMP in OS, cell proliferation, and apoptosis regulation, and most research on invasion and migration, few studies have explored whether MMP has a therapeutic target or biomarker in OS.

In addition, clusters of keywords can describe the knowledge structure of the department within the discipline and reveal research frontiers (40). Clustering analysis in the MMP-OS domain showed three clusters (**Figure 8**), partially similar to OS, proliferation, apoptosis, and invasion and migration, representing the three main directions of MMP research in OS. The role of MMP in OS has attracted attention, especially in tumor invasion and migration (41–43). However, the regulatory mechanism of MMP to promote OS invasion and metastasis is not well understood. The regulatory role of MMP in OS has not been fully elucidated, which is crucial for understanding the role of MMP in OS. It is also considered a key area of future OS research. OS often metastases pulmonary areas, making clinical treatment difficult, and MMP has a long history of research in cell invasion and migration.

References presenting intense citation burst can represent topics in a field (**Figure 10**). The most vigorous burst intensity pertained to a landmark study by Isakoff, Michael S et al. (2015) (strength = 15.93, 2018–2020), which is the most strongly cited article and the top10 co-cited reference, indicating that this study made an outstanding contribution to the field of MMP-OS (2). This review summarizes the previous treatment strategies and problems of OS and explores future clinical treatment methods. Moreover, among the top 20 references with the most substantial citations (**Figure 10**), 6 remained in the tumultuous period. These 6 references reflect the most recent MMP-OS themes and are, therefore, worthy of further discussion. According to the burst intensity, the second review (strength = 13.96) was published by Megan E Anderson et al. in Orthopedic Clinics of North America (44). The citation burst paper continued for at least six years (2016–2022). This paper reviewed the survival rates for advanced OS: prognostic factors, progression of chemotherapy and surgery, later effects of chemotherapy and surgery on survivors, and future directions. The third strongest citation burst (strength = 13.02) represented a review published in CANCER TREATMENT REVIEWS by Luetke, Anja, et al., with the burst lasting at least four years (2016–2019) (31, 44). They found the technical level of systemic OS treatment at the time by focusing on the experience of combined treatment of OS. In addition, the problems and challenges posed by tumor aggressiveness are elucidated, and the potential therapeutic direction crucial for the prognosis progression of the advanced OS is considered.

Kansara M et al. published the fourth study in “CANCER TREATMENT REVIEWS” in 2014 (11.8, 2017–2019) (45). This review summarizes features of bone development, genome-wide association studies, somatic and epigenetic characteristics, translational biology, and clinical trials of osteosarcoma. In 2018, Harrison DJ et al. published the fifth co-cited study in Expert Review of Anticancer Therapy (10.77, 2019–2022) (46). This literature reviewed several clinical trials at the time. It concluded that the prognosis of patients with OS had not improved for decades, which indicated the need to conduct research into new treatments for OS, and proposed that new drugs could be rapidly evaluated using very high-risk populations such as patients with recurrent OS to identify some innovative drugs that could be used for OS treatment.

Furthermore, the analysis showed that MMP-9 is an OS biomarker that can predict patient prognosis (47). The sixth study was published by Li H et al. in 2014 (10.73, 2015–2019). They summarized the effect of a comprehensive assessment of the effect of high MMP-9 expression on survival outcomes in patients with osteosarcoma and found that patients with high MMP-9 expression had significantly lower overall survival (47). The seventh study was published in Surgical Oncology in 2012 (9.97, 2012–2016). This review summarizes the relationship between the expression and prognosis of several matrix metalloproteinases (MMPs) in OS and specifically found that MMP-2, -8, -13, -26, and TIMP-1 are highly expressed in OS tissues. In addition to protective MMP-8, another concentrated MMP is also found in metastases, indicating that MMP and TIMP-1 can participate in OS invasion and metastasis (48). The citation burst analysis shows that OS’s etiology and diagnostic markers were summarized, and then the effect of OS treatment methods was summarized. It was concluded that advanced OS and metastatic tumors are the difficulties in the treatment of OS, but it is found that MMP is highly expressed in OS and promotes OS invasion and metastasis, which may be used as a therapeutic target and molecular marker for the refractory OS.

To precisely analyze MMP-OS research-related content, we analyzed journals, co-cited journals, and national and keyword analyses of the top most cited 100 articles.

The analysis of journals and co-cited journals (**Supplementary Tables 1**, **2**) demonstrated that MOLECULAR MEDICINE REPORTS, ONCOLOGY REPORTS, ONCOLOGY LETTERS, INTERNATIONAL JOURNAL OF ONCOLOGY, TUMOR BIOLOGY

ONCOTARGET, BIOCHEMICAL AND BIOPHYSICAL RESEARCH COMMUNICATIONS, INTERNATIONAL JOURNAL OF CLINICAL AND EXPERIMENTAL PATHOLOGY, JOURNAL OF BIOLOGICAL CHEMISTRY, EUROPEAN REVIEW FOR MEDICAL AND PHARMACOLOGICAL SCIENCES, ANTICANCER RESEARCH, CANCER RESEARCH, JOURNAL OF CELLULAR PHYSIOLOGY published the highest number of articles on MMPs-OS. In contrast, JOURNAL OF BIOLOGICAL CHEMISTRY, CANCER RESEARCH, ONCOGENE, PLoS One, CELL, PROCEEDINGS OF THE NATIONAL ACADEMY of SCIENCES OF THE UNITED STATES OF AMERICA, BIOCHEMICAL AND BIOPHYSICAL RESEARCH COMMUNICATIONS, INTERNATIONAL JOURNAL OF CANCER, Oncotarget, and CLINICAL CANCER RESEARCH acquired the most co-cited references.

The contribution of MOLECULAR MEDICINE REPORTS in MMP-OS research is noteworthy. Double-map overlay analysis of the top 100 most cited articles and all articles in the field (**Figure 11**) shows that there is only one main citation path from molecular Biology/Immunology to Molecular Biology/Genetics, suggesting that MMP-OS research highlights basic research, while traditional methods are still under-studied.

The results of a national analysis of the 100 most cited papers are consistent with the results of all articles in the field, showing that China and the United States are the largest producers. In addition, the United States is also active in cooperation with other countries, which shows that MMP-OS-related research has attracted worldwide attention (**Figure 12**).

The nation-wise analysis findings of the top 100 most-cited papers are consistent with those of all articles in the field, showing that China and the United States are the largest producers. Additionally, cooperation between the United States and other countries is active, indicating that MMP-OS-related research has attracted worldwide attention. The keyword analysis of the top 100 most-cited articles showed that they were divided into six clusters. There are common themes (OS, invasion and migration, metastasis, MMP).

The MMP-OS study initially emphasized the epidemiological characteristics and etiology of OS. The findings of this study, the study focused on the treatment strategy of OS and the treatment of advanced and metastatic tumors. It is worth noting that the expression and role of MMP in OS have gradually deepened, and it found that MMP can promote OS invasion and metastasis (**Figure 1**), which may become a new therapeutic target and marker. In addition, only three clinical trials of MMP-OS (49–51) indicate that MMP is still a long way from being applied to OS treatment.

### 4.4 Research trends

The research of MMP in OS has focused on the expression of MMP in the early stage, to the molecular mechanism affecting the occurrence and development of OS, and now focusing on the development of targeted drugs targeting MMP in OS. The dual-map overlay figure of journals related to MMP-OS and the top 100 most cited journals in the MMP-OS study showed that research published in the Molecular/Biology/Immunology journal is predominantly cited by research published in the Molecular/Biology/Genetics journal. These results also indicate that the research of MMP in OS has advanced to the field of MMP in tumor immunology.

### 4.5 The influence of MMP expression on OS prognosis

Our results suggest that many MMPs are associated with the prognosis of OS. A number of studies have been reported the molecular mechanism by which MMP regulates the prognosis of OS. On the one hand, A series of studies have reported that MMP promoted OS invasion and metastasis (41, 52–61). Specifically, MMP is an enzyme that plays an important role in the degradation of extracellular matrix components, affecting cell metabolism, survival, migration, proliferation and differentiation, excessive secretion can cause degradation to the human vascular basement membrane, accelerate the migration rate of neutral mitochondrial cells, and lead to the intensification of local inflammatory response of tissue cells (62). Some studies have shown that MMP can regulate tumor immune responses (63–66). An animal experimental study of OS showed that MMP-19 resulted in a reduced host immune response (67).

### 4.6 Future research directions

MMP is a valuable target for OS therapy in the future. However, the results of clinical trials targeting MMP drugs are not ideal, in stark contrast to their significant anti-tumor effects in numerous cell experiments and animal experiments. Many factors can contribute to these conflicting results (68). Inhibitors of MMP may be more successful if they are used in the early stages of cancer and disease. In addition, MMP inhibitors need to be more selective and make their targets more defined, thereby reducing side effects.

## 5 Limitation

This study has some inherent flaws in bibliometrics. First, collect the data and exclude some studies from the WoSCC database that are not in the WoSCC. However, WoSCC is the most commonly used scientific econometric study database; WoSCC’s data can somewhat cover most of the information. Second, all the data is obtained machine-by-machine through learning and natural language processing bibliometric tools, which can lead to biased findings among other literature research. However, compared to recent traditions Comments (69, 70), the results here are consistent and provide scholars with more objective data and insights.

## 6 Conclusions

In short, MMP-OS research is rapidly evolving, and global collaborations are active, with the United States being the main center of collaboration. Currently, the study mainly focuses on the role of MMP in OS and its therapeutic significance. The three main aspects of MMP-OS-related research include OS epidemiology, OS etiology factors, and advanced and metastatic OS treatment strategies, and MMP promotes OS invasion and metastasis and may serve as therapeutic targets. The latest hotspot is EMT, invasion, and diversion. It is worth noting that MMP inhibitors may become a hot spot for the treatment of metastatic tumors. Based on these results, the main research focus in this field is explored in this paper, and the role of MMP in OS can be studied, and the role of MMP in the treatment of metastatic OS can be explored. Our research began with systematically analyzing MMP-OS-related articles using bibliometrics and knowledge graphs. In addition, we used CiteSpace and VosViewer to analyze data to get more results from different perspectives. Unlike traditional reviews, the current study provides preliminary and objective insights into MMP-OS research. We believe that the results of this report will provide valuable references for future studies.

## Data availability statement

The original contributions presented in the study are included in the article/**Supplementary Material**. Further inquiries can be directed to the corresponding authors.

## Author contributions

Conceptualization, XW, and QZ. Methodology, XW, and SM. Software, XW, and SM. Validation, XW, and SM. Formal analysis, XW. Investigation, SM. Resources, ZW. Data curation, ZW. Writing—original draft preparation, XW. Writing—review and editing, XW. Visualization, XW. Supervision, XW. Project administration, XW. Funding acquisition, QZ. All authors contributed to the article and approved the submitted version.

## Supplementary material

Supplementary Figure 1 Association between the MMPs expression and the prognosis of OS.

Supplementary Figure 2 Association between the MMPs expression and the prognosis of OS
