# Supplementary figures and images for: Global scientific trends on matrix metalloproteinase and osteosarcoma: A bibliometric and visualized analysis
Source: Front Oncol. 2023 Feb 6;13:1064815. doi: 10.3389/fonc.2023.1064815 (PMC9939641; doi:10.3389/fonc.2023.1064815)

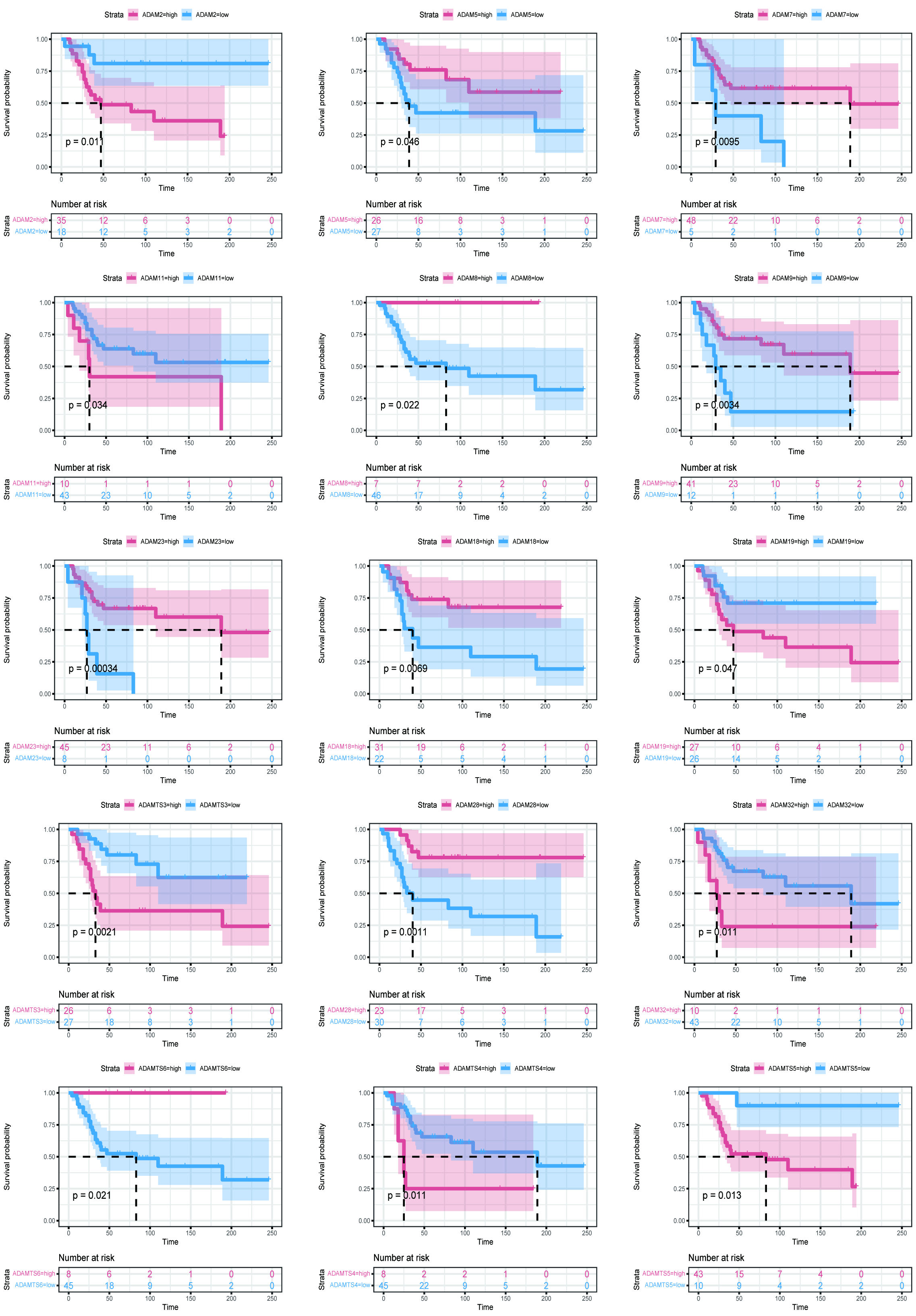

Supplement: Supplementary Figure 1 — Association between the MMPs expression and the prognosis of OS. [file Image_1.tif]

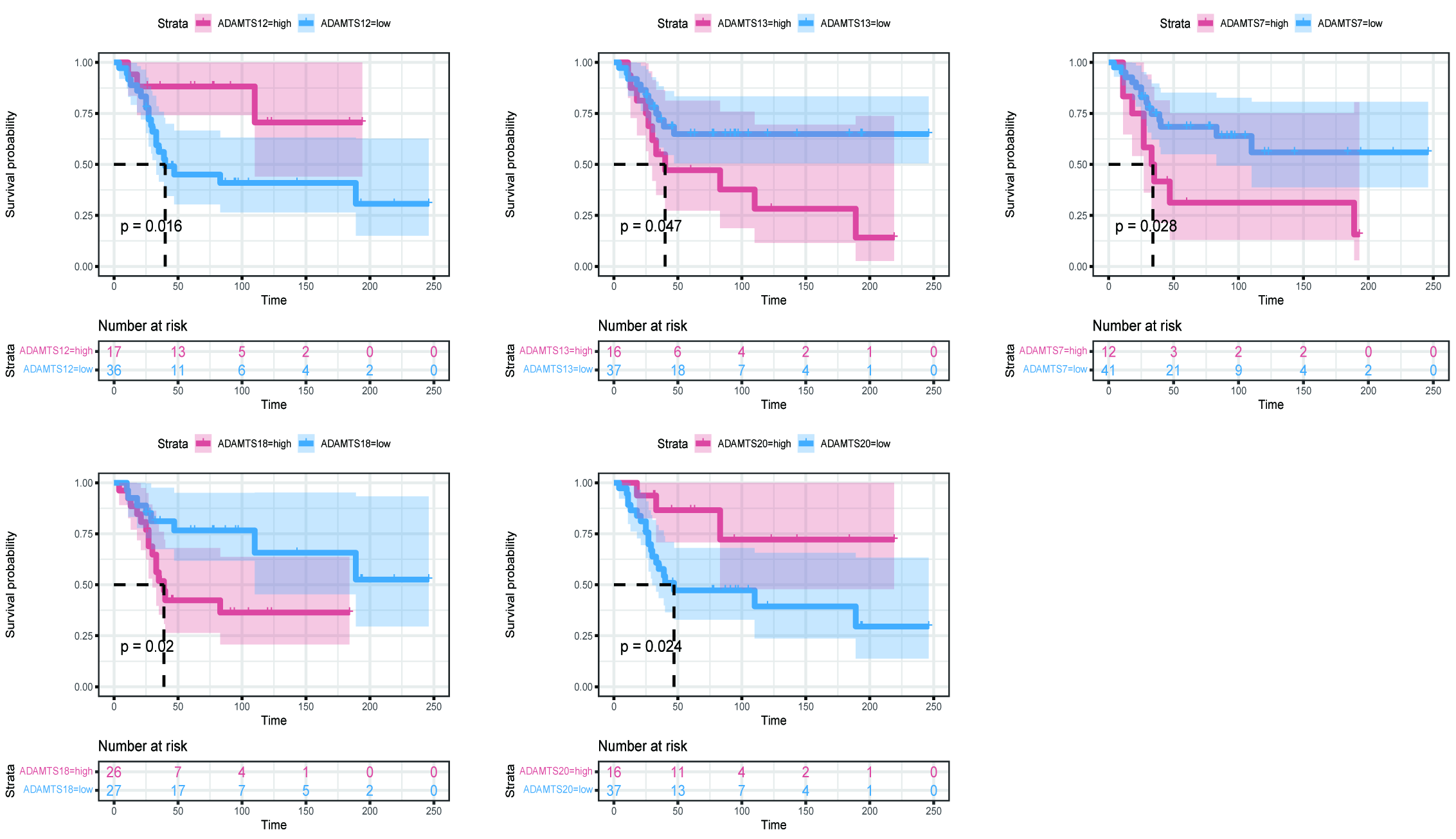

Supplement: Supplementary Figure 2 — Association between the MMPs expression and the prognosis of OS [file Image_2.tif]
